# MIL-100(Fe) Sub-Micrometric Capsules as a Dual Drug Delivery System

**DOI:** 10.3390/ijms23147670

**Published:** 2022-07-12

**Authors:** Marina Paiva Abuçafy, Regina Celia Galvao Frem, Giulia Polinario, Fernando Rogerio Pavan, Heng Zhao, Angelika Mielcarek, Cedric Boissiere, Christian Serre, Leila Aparecida Chiavacci

**Affiliations:** 1 Department of Drugs and Medicines, School of Pharmaceutical Sciences, São Paulo State University (UNESP), Araraquara 14801-902, Brazil; marina.abucafy@gmail.com; 2 Institute of Chemistry, Inorganic Chemistry, São Paulo State University (UNESP), Araraquara 14800-060, Brazil; regina.frem@unesp.br; 3 Department of Biological Sciences, School of Pharmaceutical Science, São Paulo State University (UNESP), Araraquara 14801-902, Brazil; gpolinario@gmail.com (G.P.); fernando.pavan@unesp.br (F.R.P.); 4 Institut des Materiériaux Poreux de Paris, ESPCI Paris, École Normale Supérieure, CNRS, PSL University, 75005 Paris, France; zhao.heng@ens.psl.eu (H.Z.); angelika.mielcarek@gmail.com (A.M.); 5 NanoBioMedical Centre, Adam Mickiewicz University, 61-614 Poznan, Poland; 6 Laboratoire de Chimie de la Matière Condensée de Paris (LCMCP), Collège de France, PSL, UMR 7574, Sorbonne Université, CNRS, 4 Place Jussieu, 75005 Paris, France; cedric.boissiere@upmc.fr

**Keywords:** MOF capsules, dual release systems, enzyme encapsulation

## Abstract

Nanoparticles of metal–organic frameworks (MOF NPs) are crystalline hybrid micro- or mesoporous nanomaterials that show great promise in biomedicine due to their significant drug loading ability and controlled release. Herein, we develop porous capsules from aggregate of nanoparticles of the iron carboxylate MIL-100(Fe) through a low-temperature spray-drying route. This enables the concomitant one-pot encapsulation of high loading of an antitumor drug, methotrexate, within the pores of the MOF NPs, and the collagenase enzyme (COL), inside the inter-particular mesoporous cavities, upon the formation of the capsule, enhancing tumor treatment. This association provides better control of the release of the active moieties, MTX and collagenase, in simulated body fluid conditions in comparison with the bare MOF NPs. In addition, the loaded MIL-100 capsules present, against the A-375 cancer cell line, selective toxicity nine times higher than for the normal HaCaT cells, suggesting that MTX@COL@MIL-100 capsules may have potential application in the selective treatment of cancer cells. We highlight that an appropriate level of collagenase activity remained after encapsulation using the spray dryer equipment. Therefore, this work describes a novel application of MOF-based capsules as a dual drug delivery system for cancer treatment.

## 1. Introduction

Metal–organic frameworks (MOFs) are crystalline hybrid porous materials comprised of metal ion nodes and organic linkers with exceptional structural and chemical diversity. These have gained significant attention for various applications (e.g., separation, catalysis, sensing, etc.) due to their attractive properties, which include high porosity, large surface area and structural stability [1,2,3]. In biomedicine, biocompatible MOFs are attractive candidates, particularly for drug delivery and imaging, due to their record drug loading and, in some cases, controlled delivery under body fluid conditions [4].

Biocompatible MOFs possess pore sizes typically within the micro- or mesoporous domain, e.g., within the 0.3–4 nm range, which restrict their ability to encapsulate large bioactive molecules, such as proteins. Capping of the external surface of MOF NPs is still a possibility, but such a strategy is associated both with challenges in surface modification to avoid any burst release as well as loading limitations. Therefore, to overcome this, developing new drug delivery systems (DDS) comprising MOF capsules has been proposed very recently due to their desirable properties, including large cavities, selective permeability and high stability [5,6]. This hierarchical system allows the incorporation of a large amount of a drug while enabling the loading, within the capsule, of a significant amount of macromolecules, such as proteins, carbohydrates, lipids, nucleic acids and enzymes. This enables the drug to be administered in combination with a bioactive molecule that acts in synergy, such as a drug and a protein, thereby increasing the efficiency of the treatment.

MOF capsules with a controlled chemical composition can be synthesized using several methods, including solid mold [7], liquid mold [5] and Pickering emulsion [8]. Such techniques are indeed not suitable for preparing a large amount of sub-micrometric capsules. For instance, the use of emulsion to synthesize capsules is far from being straightforward to directly prepare reproducible capsules based on MOF NPs due to the complexity of preparation using polar/nonpolar solvents and surfactants as well as stability issues. Besides these limitations, the synthesis of MOF capsules still suffers from many additional challenges, such as achieving good chemical and mechanical stability, without any coalescence as well as a robust simple and scalable production method of capsules associated with a controlled superstructure.

The spray-drying method offers many advantages to sort out these issues. This method allows the production of chemically homogeneous droplets containing pre-formed MOF NPs with no need for any additional immiscible toxic solvents, surfactants, polymers or the use of sonication [9]. Herein, we have selected spray-drying as a rapid, low-cost and scalable green route for the synthesis of MOF capsules of a sub-micrometric size with controlled hierarchical porosity. The technique has previously been used to produce dry suspensions, encapsulate biomolecules for pharmaceutical applications [10] and synthesize MOF NPs [11,12], and it is also suitable to develop a spherical hierarchical pore system from a suspension of dispersed MOF NPs to produce small-size MOF NPs capsules. Furthermore, spray-drying is a common technique used for the encapsulation of drugs and bioactive molecules in materials capable of protecting them and increasing their stability, releasing them in a controlled manner in the body, with the possibility of association with other drugs [13].

Among all the MOFs nanocarriers reported to date, the mesoporous iron trimesate MIL-100(Fe) (MIL stands for Materials from Institute Lavoisier) is one of the most promising MOFs in biomedicine due to its very high porosity, open metal sites as well as its lack of toxicity due to its bio-friendly composition and biodegradable character [14,15]. Previous studies indicated that MIL-100(Fe) NPs are stable in aqueous and ethanolic solution but degrade progressively in simulated biological media, particularly at pH 7.4 [16,17]. However, we hypothesize here that aggregating MOF NPs to form a porous capsule by spray-drying can interfere with the rate of degradation of the nanoparticles, and, thus, the release of the drugs, while enabling the enzyme to be incorporated within the additional mesopores from the capsule (interparticular and internal cavities). Moreover, the MIL-100(Fe) micronic capsules should be ideal candidates for oral or intraperitoneal administration routes but are not suitable for intravenous administration [18]. The closer system reported in the literature is a simple composite material made of ~125 nm MIL-100(Fe) submicronic particles, aggregated with carbohydrates by spray-drying [19].

Herein, we propose the synthesis and processing of MIL-100(Fe) hierarchical sub-micronic capsules as a dual drug delivery system coupling an efficient anticancer drug with an enzyme, potentializing the drug efficiency in vivo. The material reported and tested here is structurally more sophisticated than previously described spray-dried MOFs. The encapsulated drug and enzyme are localized in different porous cavities of the capsule in order not to interfere in terms of loading while slowing down their release. Methotrexate (MTX) is an antimetabolite chemotherapeutic agent specific for growing cell populations, such as tumor cells; collagenase (COL) is an enzyme that also acts as a protector against tumor progression and metastasis due to its ability to enhance degradation of the dense extracellular matrix (ECM) surrounding tumor cells. MTX was encapsulated in the mesopores of MIL-100(Fe) NPs from an aqueous solution prior to the uptake of collagenase directly into the interparticular cavities of MOFs colloids during the formation of the MIL-100 capsules by spray-drying. This resulted in the formation of MTX@COL@MIL-100 capsules that may have a potential application in the selective treatment of cancer cells.

## 2. Results

**Figure 1:**
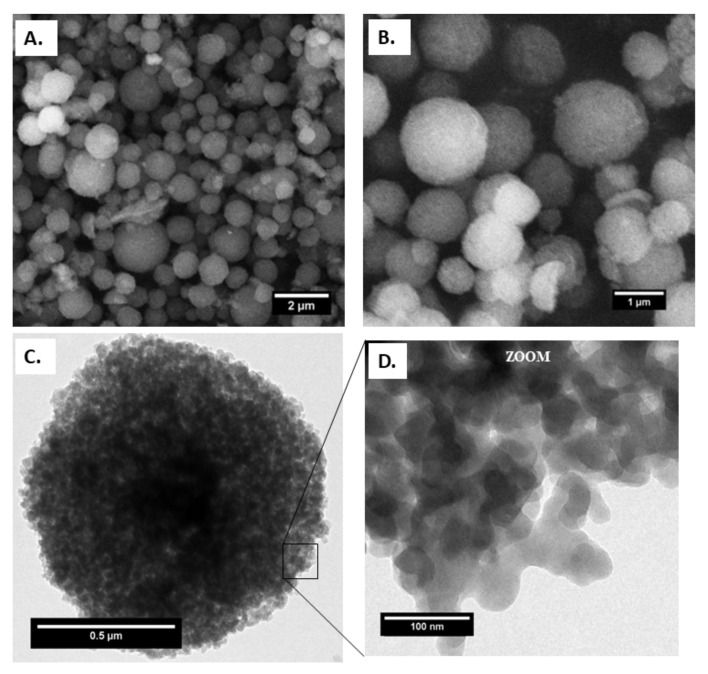
(**A**) and (**B**) field emission gun scanning electron microscopy (FEGSEM), (**C**) transmission electron microscopy (TEM) images of capsules and (**D**) MIL-100(Fe) NPs on the capsule surface.

In order to prepare the MIL-100(Fe) capsules, preformed MIL-100(Fe) NPs were synthesized separately at ambient temperature, as previously reported by our group [20]. All characteristics were, on the whole, in agreement with previous results, including a BET surface area of 1300 m^2^ g^−1^. The nanoparticles exhibited a mean diameter of 80 ± 30 nm, as determined by dynamic light scattering (DLS) (Figure S1), 66 ± 12 nm, as determined by transmission electron microscopy (TEM) (Figure 1D) associated with a ζ potential (deionized water) of −24 ± 0.6 mV, consistent with the literature [16,17]. Furthermore, the nitrogen physisorption of MIL-100(Fe) NPs exhibited BET surface area and BJH pore size distribution according to what was previously reported in the literature, as shown in Figure S2.

The resulting MIL-100(Fe) capsules preserved the crystallinity of MIL-100, as confirmed by PXRD and FTIR studies, in comparison with the pre-formed MIL-100(Fe) NPs (Figures S3 and S4). Nitrogen physisorption measurements on the capsules indicated a porosity similar to that of the bare nanoparticles, with a measured surface area of 1613 m^2^ g^−1^, with micropore and mesopore volumes of 0.64 (calculated via t-plot) and 0.85 cm^3^ g^−1^ (calculated via BET).

A complete study of the morphology and structure evolution of the MIL-100(Fe) capsule was performed by electron microscopy. The SEM and TEM images of MIL-100 capsules, shown in Figure 1, confirm the homogeneous non-hollow materials and reveal the absence of any significant cracks or defects.

Figure 1A,B show the external structure of the spray-dried capsules. SEM shows that the latter exhibit a spherical shape with an average diameter of 800 nm, as shown in Figure S5. In addition, the constitutive MIL-100(Fe) NPs exhibit the same size and morphology as the bare nanoparticles, being ca. 70 nm in diameter, as shown in Figure 1D.

**Figure 2:**
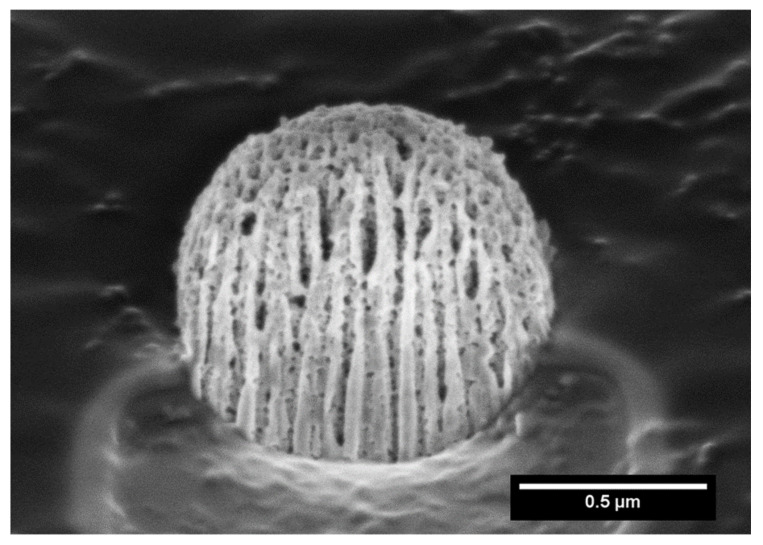
Focused ion beam scanning electron microscopy (FIB-SEM) of a MIL-100 capsule slice.

In order to further investigate the microstructure of the capsule, focused Ga^+^ ion beam (FIB) SEM analysis was carried out. Hence, FIB/SEM is an ideal combination of high-resolution nanoparticle imaging slices. A slice of the capsule was first produced, leading to a plane cutting through it, and imaging evidenced the presence of large cavities, as shown in Figure 2.

After complete characterization of the material, the drug MTX and the collagenase enzyme were incorporated into the MIL-100 capsule. The crystalline structure of the MOF remained intact after the loading experiments with little changes in the PXRD patterns in comparison with the starting nanoparticles (Figure S6A) [14].

The presence of MTX and collagenase in the structure of the capsule was further confirmed by FTIR spectroscopy (Figure S6B). The spectrum of the MTX@COL@MIL-100 capsule (blue line in Figure S6B) shows bands of the drug, such as the one at 1616 cm^−1^ attributed to the C=O stretching mode of MTX molecules. In addition, a band at 1504 cm^−1^, attributed to N-H bending of the amidic group, as well as the one of the –C–O stretching of the carboxylic group of MTX, was displaced from 1268 cm^−1^ for the encapsulated material. The IR spectrum of the MIL-100 capsules (red line in Figure S6B) was also similar to that of the MIL-100 capsule with collagenase (blue line in Figure S6B), except for the latter additional band characteristics of the enzyme, i.e., amide I at 1651 cm^−1^ (mainly from C=O stretching), amide II at 1547 cm^−1^ and amide III at 1238 cm^−1^. This confirmed the presence of collagenase in the MOF capsule, even after the washing steps.

**Figure 3:**
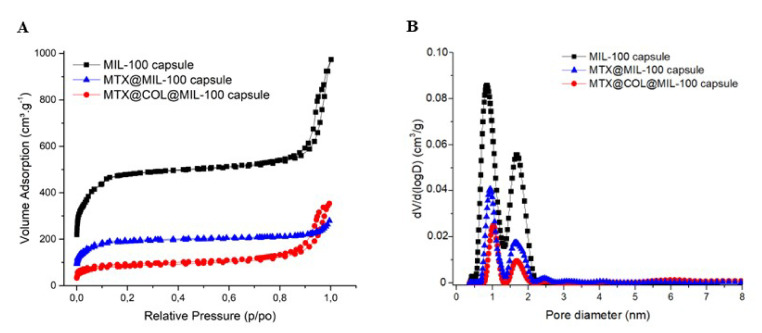
Nitrogen sorption isotherms (**A**) and pore size distribution curves (**B**) at 77K (Po = 1 bar) of MIL-100 capsules (black), MTX@MIL-100 capsules (blue) and MTX@COL@MIL-100 capsules (red).

The porosity of the capsule was also analyzed by N_2_ porosimetry and compared with bare MIL-100(Fe) nanoparticle and also before and after the encapsulation, as shown in Figure 3. Figure 3B compares the pore size distribution (PSD) of the MIL-100(Fe) capsules and the different loaded samples prepared. The two peaks correspond to the pore diameter of MIL-100(Fe) meso- and micro-sized internal cavities (≈2.4 and 1.58 nm).

**Figure 4:**
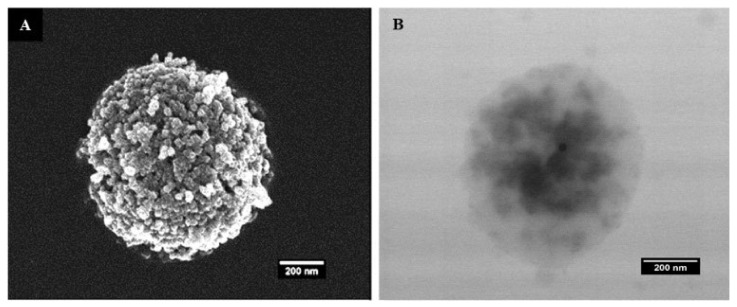
(**A**) field emission gun scanning electron microscopy (FEGSEM) and (**B**) transmission electron microscopy (TEM) images of MTX@COL@MIL-100 capsule.

The SEM and TEM micrographs of the MTX@COL@MIL-100 capsule are shown in Figure 4. The MIL-100 capsules showed well-defined particles with a homogenous density, while moderately electron-dense spots could be seen through the MTX@COL@MIL-100 capsule (Figure 4B), possibly associated with the presence of collagenase (or MTX agglomerates) inside the MOF capsules.

The MTX entrapment efficiency (EE%) in MIL-100 NPs was 50.3%. The structural integrity of the MOF was kept after encapsulation of MTX, while the residual porosity was low due to the drug loading (see characterizations in the Supplementary section).

**Figure 5:**
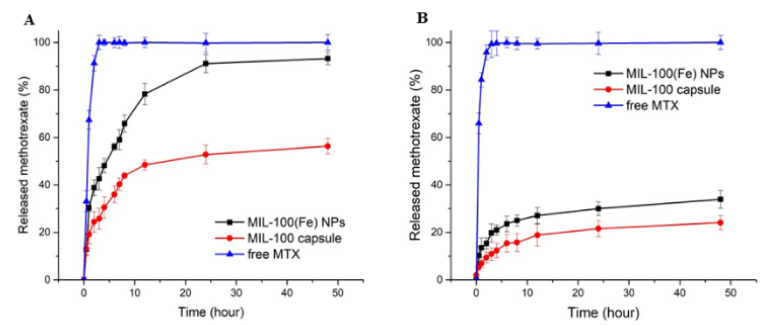
Release profiles of MTX at 37 °C from MIL-100(Fe) NPs and MIL-100 capsules in PBS at pH 7.4 (**A**) and pH 5 (**B**).

In a second iteration, MTX@MIL-100(Fe) NPs were added to a collagenase ethanolic suspension and the mixture was pumped into a spray dryer. The liquid fed into the spray dryer produced fine atomized droplets. These droplets were injected into a drying gas chamber, where vaporization occurred with outlet air temperature of 45 °C. Considering that the residence time in the drying chamber is between two and three seconds (estimated from the dry air flow rate and the volume of the drying chamber), the drying process has a small impact and should not significantly denature the collagenase [21], which was confirmed by the enzymatic activity experiment. After drying, MTX@COL@MIL-100 capsules were formed. The EE% of collagenase in the MIL-100 capsules was 91.74%, which demonstrates the high encapsulation yield of this process. We, therefore, demonstrated the ability of these MOFs capsules to be a high loading dual delivery system for active drugs and enzymes, collagenase and MTX in this case. During this step of the experiment, we carefully prepared capsules by spray-drying in a maximum period of 30 min, considering that MTX would almost not be released during this process, as shown in the MTX release curve in Figure 5.

At pH 7.4 (Figure 5A), the drug release from MTX-loaded capsules exhibits a lower degree of initial burst release when compared to the bare nanoparticles and, furthermore, the drug continues to be released from the capsule after this period. In addition, the cumulative drug release within 48 h reaches approximately 93% for the MIL-100 NPs, against ca 50% for MIL-100 capsules. Nanoparticles have very large surface area to volume ratios compared to the capsules, and, therefore, the hierarchical packing of MIL-100(Fe) colloids into capsules can slow down the MIL-100(Fe) degradation from the phosphate anions present in the medium associated with the drug release, ultimately resulting in slower MTX release. At pH 5.0 in the first 4 h, only 12% and 21% of the drug were released from the MIL-100 capsules and the bare nanoparticles, respectively, while the maximum amounts of MTX released from the MIL-100 capsules and bare nanoparticles after 48 h were only about 24% and 34%, respectively.

**Figure 6:**
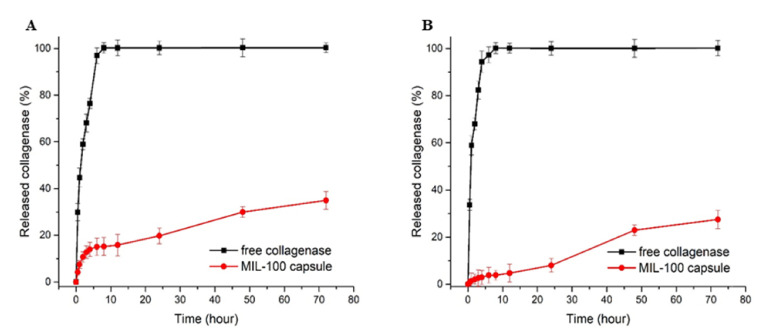
Collagenase release profiles from free enzyme and collagenase-loaded MIL-100 capsules at pH 7.4 (**A**) and pH 5 (**B**).

The collagenase release profiles from collagenase-loaded MIL-100 capsules (pre-loaded with MTX) at pH 7.4 and pH 5 are shown in Figure 6.

In detail, one can observe a tiny burst release at the early stage of the delivery, with about 15.4% of the collagenase released from the MIL-100 capsules within the first 8 h. Between 8 and 12 h, the release profile was stabilized, and, after 12 h until the end of the assay (72 h), an 18% increase in the enzyme release was observed. At pH 5.0, the initial burst release was decreased to only 3.8%, while, after 24 h, the maximum amount released was only 26.9%, which is 8% less than the value obtained at pH 7.4. The tumor tissue environment is moderately acidic compared to healthy cells; therefore, a pH-responsive drug delivery system may reduce the undesirable effects related to drug transport in blood circulation and improve anticancer drug delivery to the tumor tissue.

The kinetic MTX and COL release profiles were further fitted using the following mathematical models, using the Sigma Plot10.0 software (Systat Software, San Jose, CA, USA): Korsmeyer–Peppas, Higuchi, Hixson–Crowell, first order and Weibull. The kinetic release values are shown in Tables S3–S5. The MTX and COL release from the MIL-100 NPs and MIL-100 capsules at both pH values showed a strong correlation with the Korsmeyer–Peppas model. Values of ƞ between 0.45 and 0.89 represent cases of anomalous or non-Fickian transport.

**Figure 7:**
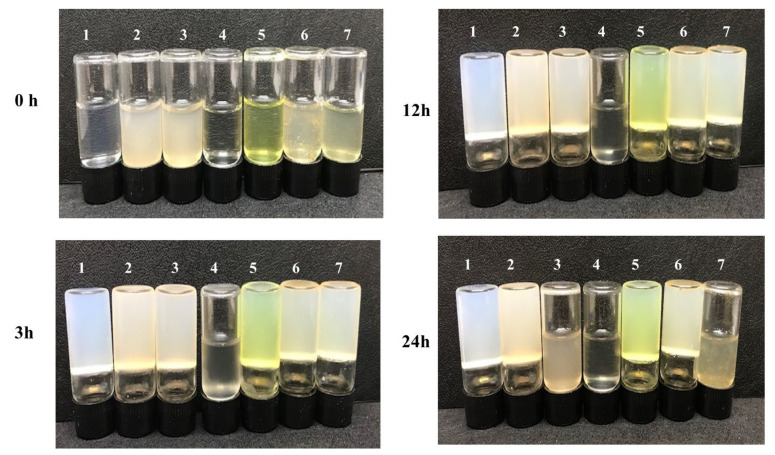
Gelatin solution cocultured with each sample at 37 °C and then stored at 4 °C for 3, 12 or 24 h; (**1**) pure gelatin; (**2**) MIL-100 capsule; (**3**) COL@MIL-100 capsule; (**4**) collagenase; (**5**) MTX; (**6**) MTX@MIL-100 capsule and (**7**) MTX@COL@MIL-100 capsule.

In vitro activity experiments were carried out to verify the properties of the dual MOF capsule carrier system. The enzymatic activity of collagenase released from the MTX@COL@MIL-100 capsule on gelatin degradation was first measured, as shown in Figure 7. It was found that pure gelatin solidifies back to a hydrogel at 4 °C (group 1). In addition, gelatin could still form a steady hydrogel at low temperature even after co-incubation with empty MIL-100 capsules (group 2), free MTX (group 5) and MTX@MIL-100 capsules (group 6) for 24 h, indicating that these samples could not degrade gelatin. However, gelatin co-incubated with free collagenase (group 4) remained in a liquid state after 3 h, while the one co-incubated with COL@MIL-100 capsule (group 3) or MTX@COL@MIL-100 capsule (group 7) maintained a liquid state for between 12 and 24 h from the beginning of the assay.

To confirm the possibility of using this system for drug delivery, cytotoxicity tests were performed using the resazurin assay, which estimates the metabolic activity of living cells by the quantitative conversion of the reagent into a fluorescent indicator. These tests were carried out between concentrations of 0.586 µg/mL and 150 µg/mL, and the IC_50_ and selectivity index (SI) values obtained in this study are shown in Table 1.

All the samples revealed toxicity (IC_50_ values) in the A-375 cell line; however, for the normal cell line, HaCaT, no toxicity was observed, even at the highest concentration tested, confirming its safety. The hollow MIL-100 capsule showed an IC_50_ value of approximately 70 µg/mL for the A-375 cell line, and, after encapsulation of MTX or collagenase, the IC_50_ decreased, as expected, to 16 and 19 µg/mL, respectively. Furthermore, the IC_50_ value for the capsule with the dual encapsulation of collagenase and MTX was 13 µg/mL for the tumor cell line, in agreement with a synergetic effect of both MTX and collagenase.

## 3. Discussion

The synthesis of the MOF capsule was possible due to some intrinsic properties of MIL-100(Fe) NPs. Therefore, the negative surface charge, attributed to the free carboxylate groups from the trimesic acid linkers exposed on the outer surface of the MOF NPs, suggests that electrostatic repulsion forces helped to avoid rapid aggregation on the capsule formation process during the spray-drying.

The spray-drying method enabled the formation of hot water droplets containing the MIL-100(Fe) NPs and the drugs loaded. The process starts with the atomization of the precursor solution to form micron- and submicron-size droplets using a two-fluid nozzle (mixing fluid and air). Precursor droplets came into contact with a turbulent hot gas stream with outlet air in 45 °C, promoting a rapid solvent evaporation. In each droplet, all the constituents move both by diffusion and convection, while the droplet diameter decreases with evaporation, resulting in the rapid formation of capsules made of MIL-100(Fe) NPs stacked concentrically with each other. To benchmark our spray-drying technique with results previously reported by some of us [11], we initially focused on the most important input parameters, such as the solid concentration (1 mg·mL^−1^), the drying gas inlet temperature (80 °C), the solvent type (ethanol) and the drying gas flow rate (0.05 mL·min^−1^), aiming to form nano-micrometric and spherical capsules. The adjustment in the gas inlet temperature influences the evaporation rate, thus transfer of suspended constituents from the surface to the deepest area inside the drop, and this can create hollow spaces in the particle. In this study, the temperature used, outlet temperature of 45 °C, was judiciously low, high enough to evaporate the aqueous suspension and low enough for limiting the degradation of the enzyme [22].

The TEM and SEM images, as shown Figure 1 and Figure 4, confirm that the selected low-temperature spray-drying conditions enabled the gentle aggregation of pre-formed MIL-100(Fe) NPs without using emulsion, most commonly used in the literature [12,23].

We highlight here that this processing procedure produces non-hollow particles, which allows adjusting the final particle size simply by adjusting the concentration of the MIL-100 nanoparticle suspension. This point is of crucial importance for it allows easily tuning the aerodynamic diameter of the particles if optimized lung delivery is targeted, which is not the case in the article previously reported, in which dextran and alpha cyclodextrine/MIL-100 spray-dried particles, as reported previously, are hollow and, thus, for which the density is not a priori predictable and changes with the particle diameter [19]. In addition, nitrogen physisorption measurements, shown in Figure 3 and S2, confirmed that the spray-drying process does not affect the textural quality of the previously synthesized MOFs. The N_2_ sorption isotherm of the MIL-100 capsules is associated with a combination of the intrinsic micro/mesoporosity of the bare nanoparticles and the additional interparticular porosity of the voids between the nanoparticles aggregates delimiting the capsules. The FIB/SEM of the capsule slice also showed large cavities (Figure 2), which are of a strong interest for carrying large active biomolecules, such as collagenase.

These cavities, combined with the intrinsic micro/mesopores from the MIL-100 constitutive particles, comprise a promising hierarchical pores carrier for the controlled delivery of a large variety of drugs and biomolecules to treat cancer. As a proof of concept, we first selected MTX, a folate antimetabolite and potent anticancer molecule that interacts with the reduced folate carrier and the folate receptor, which is overexpressed on the surface of several types of cancer cells [24,25]. Furthermore, to take advantage of the large voids from the capsule, we also selected the COL, which is part of a group of enzymes called proteases that have the ability to hydrolyze peptide bonds. These enzymes are used in cancer therapies to degrade extracellular proteins and tumor cell growth factors, thereby preventing tumor progression and metastasis [26]. Therefore, this MOF-based capsule system with a multi-modal porosity represents a promising strategy to associate two bioactive molecules into a single material, known as a dual delivery system [27,28].

The MTX encapsulation was higher than what has been previously reported in the literature by other nanocarriers. For example, Trotta et al. reported an EE% in liposomes of 39.6%, while another group showed an EE% of only 10.2% for silver nanoparticles coated with PEG [29]. These results confirm the excellent capacity of MIL-100 NPs to incorporate a large amount of this drug. The structural integrity of the MOF was maintained after encapsulation of MTX, while the residual porosity was low due to the drug loading (Figure S6 and Table S2). Besides that, the high encapsulation of collagenase in the MIL-100 capsules demonstrates the ability of these MOFs capsules to be a high loading dual delivery system for active drugs and enzymes, collagenase and MTX in this case.

The porosity of the capsule analyzed by N_2_ porosimetry properties (please see Table S2) showed a decrease in the specific surface area of the capsule, which can be justified by the incorporation of a large amount of MTX and collagenase. Besides that, the incorporation of the drug and collagenase led to an even stronger decrease in pore volume, as expected.

The SEM and TEM micrographs of the MTX@COL@MIL-100 capsule, presented in Figure 4, show that the spherical morphologies of the capsules were maintained after drug and enzyme encapsulation.

In drug release experiments, whose results are shown in Figure 5 and Figure 6, the higher release in MIL-100 NPs is justified by large surface area to volume ratios compared to the capsules, and, therefore, the hierarchical packing of MIL-100(Fe) colloids into capsules can slow down the MIL-100(Fe) degradation from the phosphate anions present in the medium associated with the drug release, ultimately resulting in slower MTX release. At pH 5.0, an even slower and lower MTX release rate is observed in comparison with the profile at pH 7.4, as expected from these iron MOFs that are more stable under acidic conditions. Briefly, the amount of drug released in a more acidic environment was approximately half the amount of drug released at pH 7.4. This is mainly due to the greater stability of MIL-100 in an acidic medium, which is in line with previous studies that showed that MOF NPs progressively release their ligand in neutral PBS, whereas, in acidic conditions, the degradation kinetics was much lower [16,17]. Therefore, these results highlight that MTX is released slowly from the MIL-100 capsules in a simulated physiological medium, in favor of their use as advanced controlled release systems.

Noteworthily, in both cases, at pH 7.4 and 5, the release profiles are, on the whole, in agreement with a very slow release of collagenase. Further, the collagenase is in the interparticle space MIL-100(NPs), suggesting that it may be located more superficially or deeper in the capsule. The initial release can tentatively be attributed to the presence of the enzyme in cavities close to the outer surface of the capsule. The release profile stabilizing between 8 and 12 h of the experiment is due to the enzyme located within the internal cavities of the capsules. After 12 h, the increase in the enzyme release is in agreement with the slow diffusion of the enzyme through the cavities of the capsule. At pH 5.0, the kinetics of release was overall slower than the one observed at pH 7.4. Such a difference in release profiles could be due to the greater stability of the constitutive MOF (and, thus, MOF capsule) in the acidic medium, releasing only a small amount of collagenase present inside the MIL-100 capsule and more pronounced degradation in a neutral PBS, which, consequently, led to a higher collagenase release [17]. This longer sustained release of the enzyme, which fits well with the one of MTX under the same conditions, can be explained by its localization within the core of the capsule. It is known that collagenase, as a large biomolecule 11.5 nm in diameter [30], cannot diffuse through the MIL-100(Fe) microporous windows; therefore, to be released, it must diffuse through the routes of inter-particular spaces.

The quantitative interpretation of the in vitro release values obtained is facilitated by using equations that mathematically translate the release profile according to some parameters, as shown in Tables S3–S5. Mathematical modelling has been an important tool regarding the physical–chemical processes involved during the drug release process in the studied matrix. The MTX and COL release from the MIL-100 NPs and MIL-100 capsules showed a correlation with the Korsmeyer–Peppas model and with anomalous or non-Fickian transport. The non-Fickian diffusion includes more than one physical–chemical process in release mechanisms simultaneously, such as swelling, diffusion and system dissolution [31]. Thus, the release of MTX must occur by diffusion and erosion as the MIL-100 NPs degrade in the medium, likely to be associated with a change in their surface chemistry with the attack of the phosphate present in the medium [17].

Finally, in vitro collagenase activity experiments, whose photos are presented in Figure 7, showed that the MOF capsule carrier system with COL, as it was co-incubated with gelatin, maintained a liquid state, indicating that the gelatin was degraded, which demonstrated that the collagenase maintained an appropriate level of enzymatic activity after encapsulation to be used in association with the MTX to be used in antitumor application.

The cytotoxicity test results, shown in Table 1, highlight the strong selectivity of our system for the tumor cell line used in the experiment. The hollow MIL-100 capsule already showed tumor selectivity; however, it increased by nine times after MTX encapsulation and seven times after collagenase encapsulation. In addition, the SI assay showed that the multidrug incorporation of MTX and collagenase led to an even higher selectivity (×11), suggesting that the MTX@COL@MIL-100 capsule may have a potential role in the selective treatment of cancer cells. Therefore, such dual drug delivery systems developed based on MIL-100 capsules are promising candidates for multimodal antitumor treatment.

## 4. Materials and Methods

### 4.1. Production of MIL-100(Fe) Capsules by Spray-Drying

The spray-drying procedure was conducted on a laboratory scale (Mini Spray Dryer B290) with two concentric fluid nozzles (fluid and air). The key parameters controlling the final particle size are the spray mesh size, concentration of solids, pump flow and aspirator and inlet temperature [13]. The pre-formed MIL-100(Fe) NP suspension was injected simultaneously with hot air in the spray-drying apparatus with a nozzle orifice diameter of 0.5 mm. After a comprehensive investigation and studies previously carried out by some of us, the parameter settings were established, specifically a suspension concentration of 1 mg·mL^−1^, inlet air temperature of 80 °C, outlet air temperature 45 °C, dry air pressure of 50 bar and pump flow rate 0.05 mL·min^−1^.

### 4.2. Characterization

Powder X-ray diffraction patterns (PXRD) were obtained by a Bruker D8 Advance diffractometer (0–2ϴ) using Cu K_α_ radiation (λ = 1.5418 Å). Transmission IR spectra were recorded in the range of 400–4000 cm^−1^ on a Nicolet iS5 iD7 ATR spectrometer. N_2_ sorption isotherms were obtained at 77 K using an ASAP porosimeter (accelerated surface area and porosimetry system, model 2013; Micromeritic) connected to a computer. Prior to the analysis, samples were evacuated at 120 °C under primary vacuum. Brunauer–Emmett–Teller (BET) surface area and pore volume were estimated at a relative pressure ranging from 0.05 and 0.25. Field emission gun scanning electron microscopy (FEGSEM) observations were obtained by using a TopconSM-300 microscope with a secondary electron detector and an electron acceleration voltage of 10 kV. Dual beam focused ion beam scanning electron microscope (FIB/SEM) images were obtained by using an FEI microscope (HELIOS NANOLAB 600i) with a gallium ion column. HRTEM images were recorded on a FEI Tecnai G^2^ F20 transmission electron microscope (TEM) operating at 200 kV, equipped with an electron scattering transmission emission. Samples were prepared by the deposition of one droplet of colloidal suspension onto a carbon-coated copper grid, which was left to air dry.

### 4.3. Methotrexate Encapsulation and Release

Methotrexate was loaded by soaking 60 mg of MIL-100 NPs in 40 mL of an aqueous solution of MTX (1 mg·mL^−1^) under constant stirring at 37 °C overnight. The suspension was then centrifuged (14,000 rpm, 10 min) and the supernatant was removed. The solid was washed two times with water, recovered by centrifugation (14,000 rpm, 10 min) and dried under vacuum overnight. The drug in the supernatant was measured in triplicate by UV–Vis spectroscopy (Cary 60 Spectrophotometer; Agilent, Australia) at 306 nm. The entrapment efficiency (EE%) was calculated according to Equation (1).

(1) $$\mathrm{Entrapment}\;\mathrm{efficiency}\;\left(\mathrm{EE}\%\right)=\frac{\mathrm{weight}\;\mathrm{of}\;\mathrm{drug}\;\mathrm{in}\;\mathrm{MIL}-100\;\mathrm{nanoparticles}}{\mathrm{weight}\;\mathrm{of}\;\mathrm{drug}\;\mathrm{fed}\;\mathrm{initially}}\;\times \;100\;$$

The MTX release profiles were obtained by using simulated physiological media (phosphate buffered saline (PBS) solution 0.1 M) at pH 5.0 and pH 7.4, representing the cancer environment and the physiological pH, respectively. MTX@MIL-100(Fe) NPs and MTX@MIL-100 capsules (10 mg) were suspended in PBS solution (10 mL) and incubated at 37 °C under constant stirring, prepared in triplicate. Samples were removed after 0.5, 1, 2, 3, 4, 6, 7, 8, 12, 24 and 48 h, and an identical volume of fresh PBS was immediately replaced, keeping up the MTX concentration far from saturation.

The release of MTX was quantified in triplicate by UV–Vis spectroscopy (Cary 60 Spectrophotometer; Agilent, Sidney, Australia) at 306 nm.

### 4.4. Collagenase Encapsulation and Release

Collagenase was loaded into the capsule through a spray-drying technique. Collagenase (10 mg) was added into 50 mL of an MTX@MIL-100 capsule ethanolic suspension (1 mg·mL^−1^). The mixture was quickly subjected to spray-drying using the parameters described in Section 4.1. Low-temperature dry air was used for limiting enzyme denaturation. After obtaining the MTX@col@MIL-100 capsules by spray-drying, the material was washed three times with water to remove any free collagenase present at the outer surface of the capsules. The presence of collagenase in the supernatant was determined in triplicate through UV–Vis spectroscopy (Cary 60 Spectrophotometer; Agilent, Australia) at 258 nm.

The release of collagenase was studied by suspending 10 mg of the drug-loaded MOF capsule powder in 30 mL of PBS (pH 7.4 and pH 5). This suspension (in triplicate) was kept under constant stirring for 48 h at 37 °C. At each time point, a 0.5-mL aliquot of supernatant was recovered following centrifugation (14,000 rpm, 10 min) and replaced with the same volume of fresh PBS, keeping up the total volume of solution. The release of collagenase was quantified in triplicate by UV–Vis spectroscopy (Cary 60 Spectrophotometer; Agilent, Australia) at 258 nm.

The in vitro release data were analyzed according to various kinetic models, including zero order, first order, Korsmeywer–Peppas and Huguchi models, to determine the most appropriate release models that described the release patterns of the drug and enzyme. Model selection was based on the highest correlation coefficient (r) of the assessed parameters, as suggested by Burnham and Anderson (Table S1) [32].

### 4.5. Enzymatic Activity

The enzymatic activity of collagenase after encapsulation within the MOF capsules was investigated according to the procedure previously described by Wang et al. (2018) [33]. First, 30 mg/mL of an aqueous solution of gelatin type B (from bovine Sigma-Aldrich G9391) was prepared at 37 °C. The gelatin solution was divided into seven groups: pure gelatin, MIL-100 capsules, free collagenase, free MTX, MTX@MIL-100 capsules, col@MIL-100 capsules and MTX@col@MIL-100 capsules. The collagenase concentration was equal in all samples, based on the amount encapsulated in the MOF capsules. For each time interval (3, 12 and 24 h), the sample was stored at 4 °C for 30 min before an image was obtained using a digital camera.

### 4.6. In Vitro Toxicity Assay

The cell viability of HaCaT (human keratinocytes) and A-375 (human melanoma) cell lines was determined by resazurin redox assay according to the procedure described by Pagé and coauthors [22] and adapted by Pavan and collaborators [34]. Cells were cultured in DMEM-HG supplemented with 10% FBS (Gibco) and 1% antibiotic gentamicin sulfate (50 mg/L) and antimycotic solution, amphotericin B (2 mg/L), at 37 °C, with 5% CO_2_ and 95% humidity. Cells were seeded into 96-well microplates (TPP) to achieve a concentration of 1 × 10^6^ cells/well, then incubated under standard culture conditions for 24 h to allow cell adhesion. The free MTX, MIL-100 capsule, MTX@MIL-100 capsule, collagenase, collagenase@MIL-100 capsule and MTX@collagenase@MIL-100 capsule treatments were prepared and incubated in serial dilutions starting at a concentration of 150 µg/mL. The positive control consisted of wells containing cells and the negative control consisted of only culture medium. The microplates were again incubated for 24 h. After this period, the culture medium was removed and 50 µL of 0.01% resazurin was added. The plates were again incubated for 2 h, then the fluorescence was read in a Synergy H1 microplate reader (excitation at 530 nm and emission at 590 nm). The resulting fluorescence signals were normalized to the fluorescence signal of the negative control. The IC_50_ was defined as the highest concentration that allowed 50% cell viability. All experimental points were assessed in duplicate, and bars represented the standard error of the mean.

## 5. Conclusions

Spherical metal–organic capsules, based on MOF MIL-100(Fe) capsules, were successfully synthesized through a gentle green and scalable low-temperature spray-drying process, starting from pre-formed small-size nanoparticles (<100 nm). The sub-micron-size (800 nm) capsules revealed good stability and large internal voids (30 nm), which combined to the intrinsic porous character of the MOF NPs, making them promising as a dual drug carrier system. The capsules could successfully concomitantly encapsulate a high loading of the anticancer drug MTX and collagenase associated with a controlled release in PBS over a few days, slower than what was achieved for the bare nanoparticles. The loaded MIL-100 capsule was found to be safe when applied to normal cells, while the drug loaded capsules showed a synergetic effect between MTX and collagenase, known to enhance the degradation of the dense extracellular matrix (ECM) around tumor cells through in vitro activity tests. This confirms the high potential of such porous MOF capsules as multimodal antitumor therapies.

## Figures and Tables

**Table 1:** Results of the IC_50_ (µg/mL) and selectivity index (SI).

Sample	IC50		SI
	A-375	HaCaT	HaCaT/A-375
MTX	10.37 ± 5.1	>150.00 ± 0.0	>14.46
MIL-100 capsule	69.33 ± 5.5	>150.00 ± 0.0	>2.16
MTX@MIL-100 capsule	16.29 ± 2.0	>150.00 ± 0.0	>9.21
MTX@COL@MIL-100 capsule	13.06 ± 7.9	>150.00 ± 0.0	>11.48
COL@MIL-100 capsule	18.99 ± 14.7	>150.00 ± 0.0	>7.90
Collagenase	>150.00 ± 0.0	>150.00 ± 0.0	1
DMSO	>150.00 ± 0.0	>150.00 ± 0.0	1

## Data Availability

Not applicable.

## Supplementary Materials

The following supporting information can be downloaded at: https://www.mdpi.com/article/10.3390/ijms23147670/s1.

## References

[1] Hoskins B.F., Robson R. (1989). Infinite Polymeric Frameworks Consisting of Three Dimensionally Linked Rod-like Segments. J. Am. Chem. Soc..

[2] Bowen R.J., Fernandes M.A., Hitchcock P.B., Lappert M.F., Layh M. (2002). Synthesis and Crystal Structures of Novel 1-Aza-2-Silacyclobut-3-Enes. J. Chem. Soc. Dalton Trans..

[3] Yaghi O.M., Li G., Li H. (1995). Selective Binding and Removal of Guests in a Microporous Metal–Organic Framework. Nature.

[4] Horcajada P., Gref R., Baati T., Allan P.K., Maurin G., Couvreur P., Ferey G., Morris R.E., Serre C. (2011). Metal–Organic Frameworks in Biomedicine. Chem. Rev..

[5] Ameloot R., Vermoortele F., Vanhove W., Roeffaers M.B.J., Sels B.F., De Vos D.E. (2011). Interfacial Synthesis of Hollow Metal–Organic Framework Capsules Demonstrating Selective Permeability. Nat. Chem..

[6] Shi J., Zhang L., Jiang Z. (2011). Facile Construction of Multicompartment Multienzyme System through Layer-by-Layer Self-Assembly and Biomimetic Mineralization. ACS Appl. Mater. Interfaces.

[7] Guo J., Ping Y., Ejima H., Alt K., Meissner M., Richardson J.J., Yan Y., Peter K., von Elverfeldt D., Hagemeyer C.E. (2014). Engineering Multifunctional Capsules through the Assembly of Metal–Phenolic Networks. Angew. Chem. Int. Ed..

[8] Bian Z., Xu J., Zhang S., Zhu X., Liu H., Hu J. (2015). Interfacial Growth of Metal Organic Framework/Graphite Oxide Composites through Pickering Emulsion and Their CO_2_ Capture Performance in the Presence of Humidity. Langmuir.

[9] Sosnik A., Seremeta K.P. (2015). Advantages and Challenges of the Spray-Drying Technology for the Production of Pure Drug Particles and Drug-Loaded Polymeric Carriers. Adv. Colloid Interface Sci..

[10] Ré M. (2006). Formulating Drug Delivery Systems by Spray Drying. Dry. Technol..

[11] Garcia Marquez A., Horcajada P., Grosso D., Ferey G., Serre C., Sanchez C., Boissiere C. (2013). Green Scalable Aerosol Synthesis of Porous Metal-Organic Frameworks. Chem. Commun..

[12] Carné-Sánchez A., Imaz I., Cano-Sarabia M., Maspoch D. (2013). A Spray-Drying Strategy for Synthesis of Nanoscale Metal–Organic Frameworks and Their Assembly into Hollow Superstructures. Nat. Chem..

[13] Arpagaus C., Collenberg A., Rütti D., Assadpour E., Jafari S.M. (2018). Nano Spray Drying for Encapsulation of Pharmaceuticals. Int. J. Pharm..

[14] Horcajada P., Surblé S., Serre C., Hong D.Y., Seo Y.K., Chang J.S., Grenèche J.M., Margiolaki I., Férey G. (2007). Synthesis and Catalytic Properties of MIL-100(Fe), an Iron(III) Carboxylate with Large Pores. Chem. Commun..

[15] Horcajada P., Chalati T., Serre C., Gillet B., Sebrie C., Baati T., Eubank J.F., Heurtaux D., Clayette P., Kreuz C. (2010). Porous Metal–Organic-Framework Nanoscale Carriers as a Potential Platform for Drug Delivery and Imaging. Nat. Mater..

[16] Bellido E., Guillevic M., Hidalgo T., Santander-Ortega M.J., Serre C., Horcajada P. (2014). Understanding the Colloidal Stability of the Mesoporous MIL-100(Fe) Nanoparticles in Physiological Media. Langmuir.

[17] Christodoulou I., Bourguignon T., Li X., Patriarche G., Serre C., Marli C., Gref R. (2021). Degradation Mechanism of Porous Metal-Organic Frameworks by In Situ Atomic Force Microscopy. Nanomaterials.

[18] Dhand C., Prabhakaran M.P., Beuerman R.W., Lakshminarayanan R., Dwivedi N., Ramakrishna S. (2014). Role of Size of Drug Delivery Carriers for Pulmonary and Intravenous Administration with Emphasis on Cancer Therapeutics and Lung-Targeted Drug Delivery. RSC Adv..

[19] Fernández-Paz C., Rojas S., Salcedo-Abraira P., Remuñán-López C., Horcajada P. (2020). Biological and Medical Applications of Materials and Interfaces Metal-Organic Framework Microspheres Formulation for Pulmonary Administration Metal-Organic Framework Microspheres Formulation for Pulmonary. ACS Appl. Mater. Interfaces.

[20] Panchal M., Nouar F., Serre C., Benzaqui M., Sene S., Steunou N., Giménez Marqués M. (2018). Low Temperature Process for the Synthesis of Mof Carboxylate Nanoparticles. U.S. Patent.

[21] Collagenase-Creative Enzymes. https://www.creative-enzymes.com/similar/collagenase_148.html.

[22] Page B., Page M., Noel C. (1993). A New Fluorometric Assay for Cytotoxicity Measurements In Vitro. Int. J. Oncol..

[23] de Souza Lima R., Ré M.I., Arlabosse P. (2020). Drying Droplet as a Template for Solid Formation: A Review. Powder Technol..

[24] Li Y., Lin J., Wu H., Jia M., Yuan C., Chang Y. (2014). Novel Methotrexate Prodrug-Targeted Drug Delivery System Based on PEG—Lipid—PLA Hybrid Nanoparticles for Enhanced Anticancer e Ffi Cacy and Reduced Toxicity of Mitomycin C. J. Chem. B.

[25] Chatterjee M., Maity R., Das S., Mahata N., Basu B., Chanda N. (2020). Electrospray Based Fluorescent Nanoparticle Synthesis from Pyrene Butyric Acid-Functionalized Poly (D, L-Lactide-Co-Glycolide) Polymer for the Efficient Delivery of Anticancer Drug and Self-Monitoring Its Effect in the Drug-Resistant Breast Cancer Cells. Mater. Adv..

[26] Soria-Valles C., Gutiérrez-Fernández A., Guiu M., Mari B., Fueyo A., Gomis R.R., López-Otín C. (2019). Correction: The Anti-Metastatic Activity of Collagenase-2 in Breast Cancer Cells Is Mediated by a Signaling Pathway Involving Decorin and MiR-21. Oncogene.

[27] Jang B., Kwon H., Katila P., Lee S.J., Lee H. (2016). Dual Delivery of Biological Therapeutics for Multimodal and Synergistic Cancer Therapies. Adv. Drug Deliv. Rev..

[28] Abánades Lázaro I., Wells C.J.R., Forgan R.S. (2020). Multivariate Modulation of the Zr MOF UiO-66 for Defect-Controlled Combination Anticancer Drug Delivery. Angew. Chem. Int. Ed..

[29] Trotta M., Peira E., Carlotti M.E., Gallarate M. (2004). Deformable Liposomes for Dermal Administration of Methotrexate. Int. J. Pharm..

[30] Eckhard U., Schönauer E., Nüss D., Brandstetter H. (2012). Europe PMC Funders Group. Structure of Collagenase G Reveals a Chew and Digest Mechanism of Bacterial Collagenolysis. Nat. Struct. Mol. Biol..

[31] Korsmeyer R.W., Peppas N.A. (1992). Macromolecular and Modeling Aspects of Swelling Controlled System. Control. Drug Deliv. Syst..

[32] Burnham K.P., Anderson D.R. (2002). A Practical Information-Theoretic Approach. Model Selection Multimodel Inference.

[33] Wang X., Luo J., He L., Cheng X., Yan G., Wang J., Tang R. (2018). Hybrid PH-Sensitive Nanogels Surface-Functionalized with Collagenase for Enhanced Tumor Penetration. J. Colloid Interface Sci..

[34] Pavan F.R., da S. Maia P.I., Leite S.R.A., Deflon V.M., Batista A.A., Sato D.N., Franzblau S.G., Leite C.Q.F. (2010). Thiosemicarbazones, Semicarbazones, Dithiocarbazates and Hydrazide/Hydrazones: Anti-Mycobacterium Tuberculosis Activity and Cytotoxicity. Eur. J. Med. Chem..

