# Supplementary material for: MIL-100(Fe) Sub-Micrometric Capsules as a Dual Drug Delivery System
Source: Int J Mol Sci. 2022 Jul 12;23(14):7670. doi: 10.3390/ijms23147670 (PMC9324886; doi:10.3390/ijms23147670)
Supplement: Supplementary file 1 [file ijms-23-07670-s001.zip › ijms-1765334-supplementary.pdf]

International Journal of *Molecular Sciences*

MDPI

Supporting Information

MIL-100(Fe) sub-micrometric capsules as a dual drug delivery system

Marina Paiva Abuçafy, Regina Celia Galvão Frem, Giulia Polinário, Fernando Rogério Pavan, Heng Zhao, Angelika Mielcarek, Cédric Boissière*, Christian Serre*, and Leila Aparecida Chiavacci*

S1. Supplemental Experimental Procedures

Synthesis of MIL-100(Fe) NPs.

In order to prepare MIL-100 capsules, preformed MIL-100(Fe) NPs were synthesized separately in ethanolic suspension. MIL-100(Fe) NPs were synthesized according to the procedure previously reported.[1] Iron (III) nitrate nonahydrate and trimesic acid were dissolved in distilled water and keep stirring at 60 ºC during 48 h. The as-synthesized MIL-100(Fe) NPs were washed with water and ethanol. The MIL-100(Fe) NPs were stored in EtOH at a concentration of 1 $mg{\cdot}mL^{-1}$.

Dynamic light scattering (DLS)

Dynamic light scattering (DLS) size distribution was performed using a Zetasizer Nano ZS from Malvern, UK. 1 mg of MIL-100(Fe) material was placed in 1 mL of water and the sample was stirred for 5 min. The sample suspension was transferred to the quartz cuvette cell to acquire the measurements.

UV-vis spectroscopy- Calibration curve model of methotrexate.

A stock solution was prepared by dissolving 10 mg of MTX in required of 0.1 M HCl and diluting to 100 mL with ultrapure water and PBS. Aliquots were extracted from the stock to obtain diluted solutions containing MTX in the concentrations ($\mu g{\cdot}mL^{-1}$): 1.25 $\mu g{\cdot}mL^{-1}$, 2.5 $\mu g{\cdot}mL^{-1}$, 5 $\mu g{\cdot}mL^{-1}$, 7.5 $\mu g{\cdot}mL^{-1}$, 10 $\mu g{\cdot}mL^{-1}$, 12.5 $\mu g{\cdot}mL^{-1}$, 15 $\mu g{\cdot}mL^{-1}$, 17.5 $\mu g{\cdot}mL^{-1}$ and 20 $\mu g{\cdot}mL^{-1}$. The standard solutions were analyzed using an Agilent Cary 60 spectrophotometer. Absorbance spectra were collected in the range of 200 - 800 nm with a resolution of 1 nm and scan speed of 300 $nm{\cdot}min^{-1}$.

UV-vis spectroscopy- Calibration curve model of collagenase.

A stock solution of collagenase in ultrapure water and PBS was prepared in a 30 mL volumetric flask. Aliquots were extracted from the stock to obtain diluted solutions containing collagenase in the concentrations ($\mu g{\cdot}mL^{-1}$): 30 $\mu g{\cdot}mL^{-1}$, 60 $\mu g{\cdot}mL^{-1}$, 90 $\mu g{\cdot}mL^{-1}$, 120 $\mu g{\cdot}mL^{-1}$, 150 $\mu g{\cdot}mL^{-1}$ and 180 $\mu g{\cdot}mL^{-1}$. The standard solutions were analyzed using an Agilent Cary 60 spectrophotometer. Absorbance spectra were collected in the range of 200 - 800 nm with a resolution of 1 nm and scan speed of 300 $nm{\cdot}min^{-1}$.

S2. Characterization of MOFs

Table S1: Kinetic Model Equations, where Q*t* is the quantity of the drug released at time *t*, K is the release rate constant, and ɳ is the diffusional exponent that characterizes the best fitted release mechanism.

Kinetic models	Equations
Zero order	$Q_t = Q_0 - K_0 t$
First order	$\log Q_t = \log Q_0 - \frac{K_1 t}{2.303}$
Korsmeyer-Peppas	$\log Q_t = \log K + ɳ \log t$
Higuchi	$Q_t = K_h \sqrt{t}$

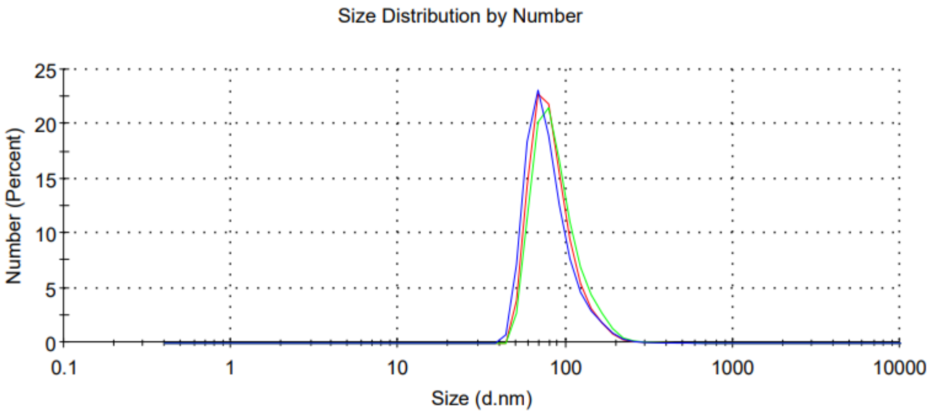

Figure S1. Dynamic light scattering (DLS) size distribution of MIL-100(Fe) NPs.

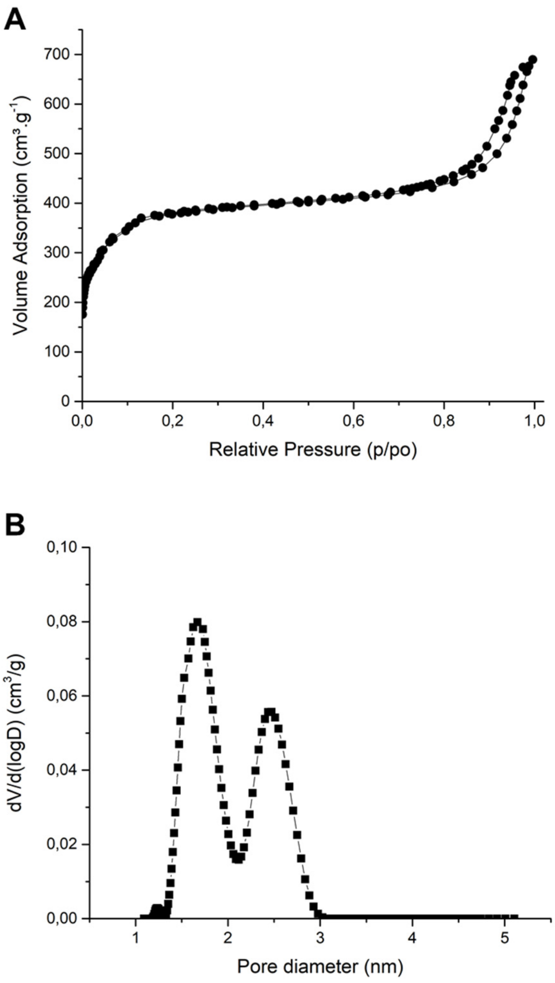

Figure S2. (A) N_2 adsorption- desorption isotherms (B) and BJH pore size distribution curves of MIL-100(Fe) NPs at 77 K (P_o = 1 atm).

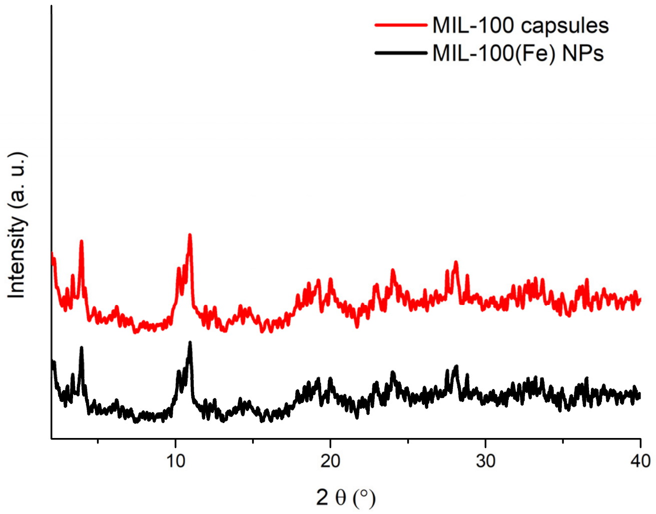

Figure S3. Powder XRD diagram of MIL-100(Fe) NPs (black line) an MIL-100 capsules (red line). The XRD patterns are in agreement with that reported in the literature.[1,2]

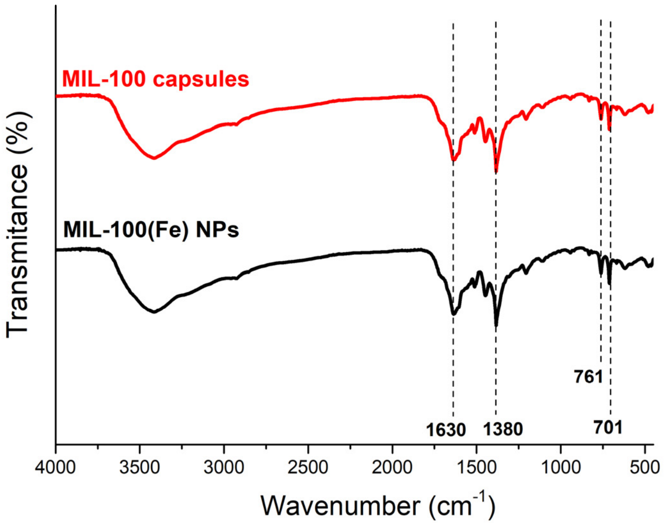

Figure S4. FT-IR spectrum of MIL-100(Fe) NPs (black line) and MIL-100 capsules (red line).

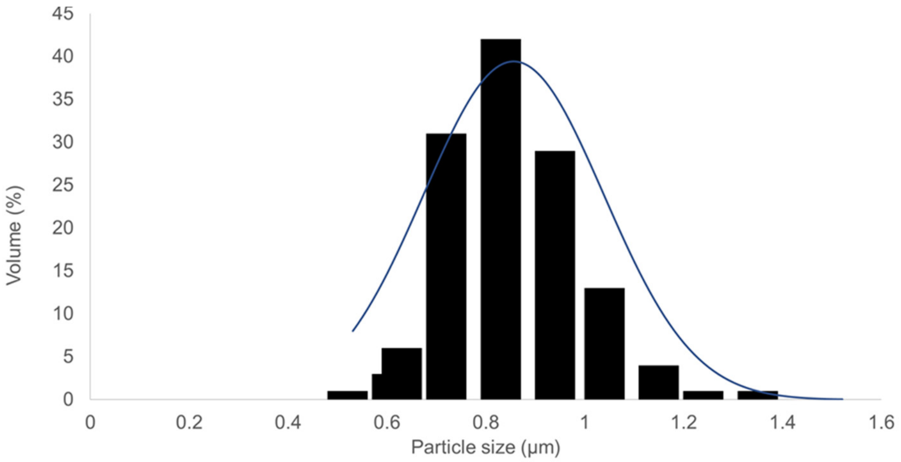

Figure S5. Particle size distribution of MIL-100 capsules.

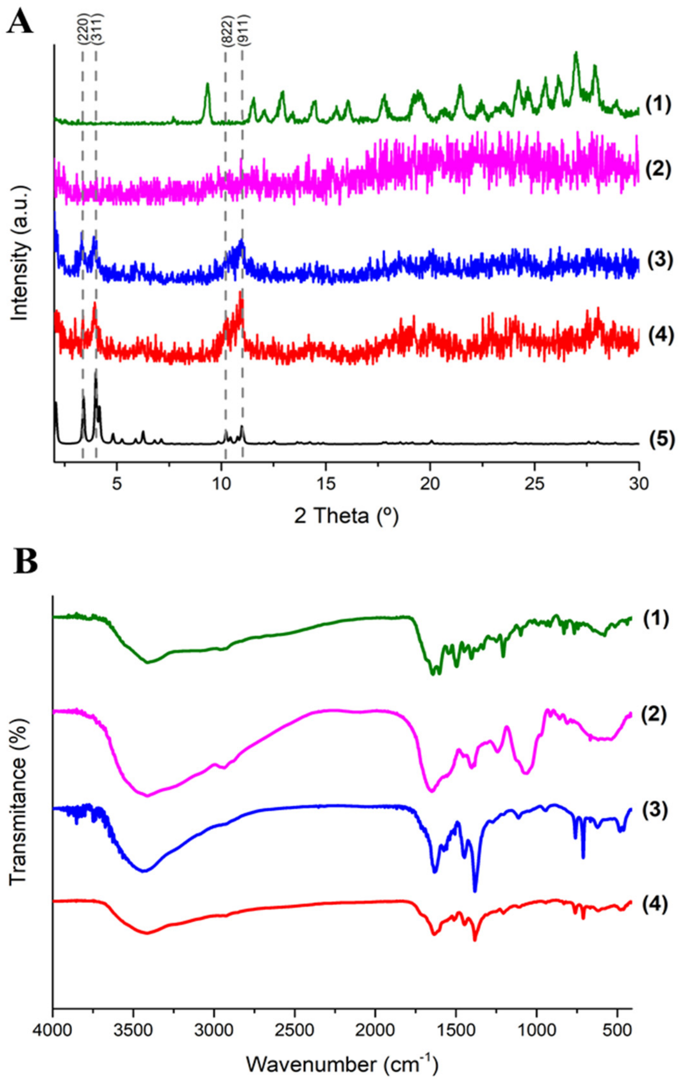

Figure S6. (A) PXRD patterns of MTX (1), collagenase (2), MTX@COL@MIL-100 capsule (3), MIL-100 capsule (4) and simulated MIL-100(Fe) NPs (5). (**B**) FTIR spectra of MTX (1), collagenase (2), MTX@COL@MIL-100 capsule (3) and MIL-100 capsule (4).

Table S2: Textural properties of MIL-100 capsules before and after MTX and collagenase encapsulation.

Sample	MIL-100 capsule	MTX@MIL-100 capsule	MTX@COL@MIL-100 capsule
BET surface area ($m^2.g^{-1}$)	1613	629	285
T-plot external surface area ($m^2.g^{-1}$)	213	125	120
Total pore volume ($cm^3.g^{-1}$)	0.85	0.32	0.21

S3. Methotrexate and Collagenase loading and release from MOF capsule

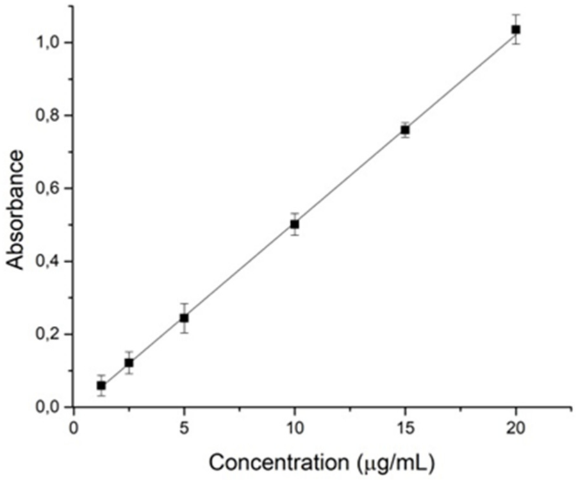

Figure S7. Calibration curve of methotrexate in water (0.1M HCl). The analytical curve obtained was: y = 0.0519x - 0.0114 and the correlation coefficient (R^2) was 0.9997, indicating linearity.

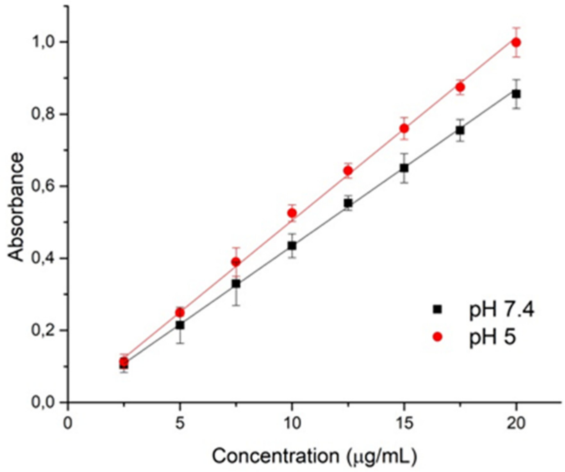

Figure S8. Calibration curve of methotrexate in PBS pH 7.4 and PBS pH 5. The analytical curve obtained in pH 7.4 was: y = 0.0439x – 0.0325 and the correlation coefficient (R^2) 0.9971, while in pH 5 was: y = 0.0506x + 0.0026 and the correlation coefficient (R^2) 0.9945.

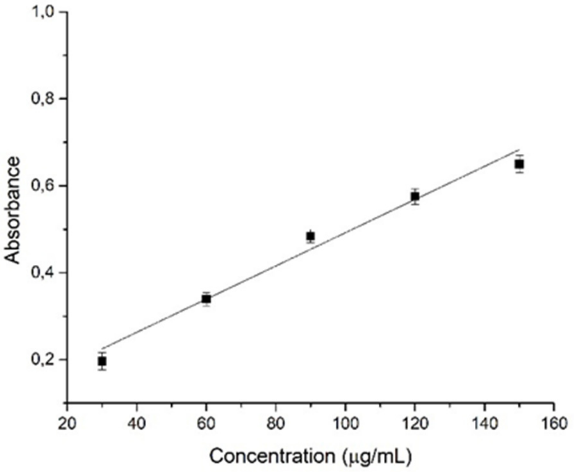

Figure S9. Calibration curve of collagenase in water. The analytical curve obtained was: y = 0.0038x + 0.0994 and the correlation coefficient (R^2) was 0.9902, indicating linearity.

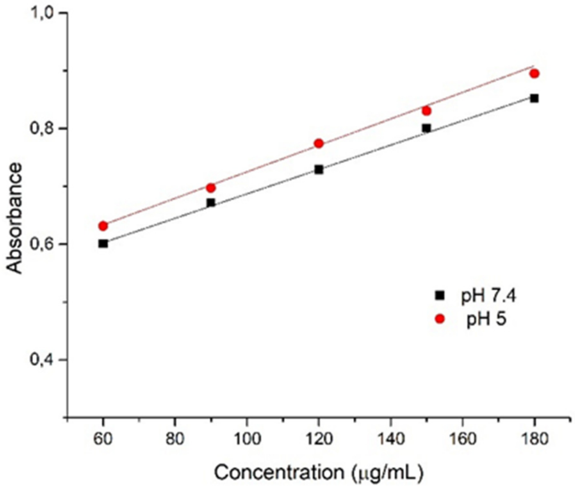

Figure S10. Calibration curve of collagenase in PBS pH 7.4 and PBS pH 5. The analytical curve obtained in pH 7.4 was: y = 0.002x + 0.4858 and the correlation coefficient (R^2) 0.9927, while in pH 5 was: y = 0.0021x + 0.5237 and the correlation coefficient (R^2) 0.9914, indicating linearity.

Table S3. MTX release kinetics from MIL-100 NPs and MIL-100 capsules in PBS pH 7.4.

	**MIL-100(Fe) NPs**		**MIL-100 capsules**	
**Mathematical model**	**r^2**	**Release exponent**	**r^2**	**Release exponent**
Korsmeyer-Peppas	0.9909	η = 0.4944	0.9989	η= 0.5468
Higuchi	0.8498		0.7076	
Hixson-crowell	0.8065		0.7203	
Primeira ordem	0.8838		0.7021	
Weibull	0.9838	b = 0.7460	0.9644	b = 0.6602

Table S4. MTX release kinetics from MIL-100(Fe) NPs and MIL-100 capsules in PBS pH 5,0.

	**MIL-100(Fe) NPs**		**MIL-100 capsules**	
**Mathematical model**	**r^2**	**Release exponent**	**r^2**	**Release exponent**
Korsmeyer-Peppas	0.9998	η= 0.4729	0.9979	η= 0.4967

Higuchi	0.9353		0.8666	
Hixson-crowell	0.6920		0.6920	
Primeira ordem	0.6854		0.6854	
Weibull	0.9710	b = 0.6870	0.9885	b = 0.7412

Table S5. Collagenase release kinetics from MIL-100 capsules in PBS pH 7.4 and pH 5,0.

	**pH 7.4**		**pH 5.0**	
**Mathematical model**	**r^2**	**Release exponent**	**r^2**	**Release exponent**
Korsmeyer-Peppas	0.9927	η = 0.8358	0.9968	η = 0.4626
Higuchi	0.9250		0.9257	
Hixson-crowell	0.3333		0.9862	
Primeira ordem	0.4052		0.9866	
Weibull	0.9842	b = 0.4699	0.9961	b = 0.7241

References

1. Horcajada, P.; Surblé, S.; Serre, C.; Hong, D.Y.; Seo, Y.K.; Chang, J.S.; Grenèche, J.M.; Margiolaki, I.; Férey, G. Synthesis and Catalytic Properties of MIL-100(Fe), an Iron(III) Carboxylate with Large Pores. *Chem. Commun.* **2007**, *100*, 2820–2822, doi:10.1039/b704325b.
2. PANCHAL, M.; NOUAR, F.; SERRE, C.; BENZAQUI, M.; SENE, S.; STEUNOU, N.; GIMÉNEZ MARQUÉS, M. Low Temperature Process for the Synthesis of Mof Carboxylate Nanoparticles 2018.
